# Thrombus or Vegetation: A Diagnostic Dilemma in Cardiac Imaging

**DOI:** 10.7759/cureus.107316

**Published:** 2026-04-18

**Authors:** Ahmed Kazi, Aniq Saleem, Abeera Akram

**Affiliations:** 1 Internal Medicine, Saint Francis Hospital, Hartford, USA; 2 Medicine, Fatima Memorial Hospital, Lahore, PAK; 3 Cardiology, University of Connecticut, Farmington, USA

**Keywords:** cancer-associated thrombosis, cardiac mass, cardiac mri, diagnostic dilemma, infective endocarditis, multimodality cardiac imaging, non-bacterial thrombotic endocarditis, thrombus, valvular lesions, vegetation

## Abstract

Cardiac masses identified on echocardiography can pose significant diagnostic challenges, particularly when differentiating between thrombi and vegetations, which require markedly different management approaches.

We present the case of a 57-year-old postmenopausal woman with a history of thymic cancer and recent pulmonary embolism (PE), who presented with abdominal pain, weight loss, and laboratory findings consistent with disseminated intravascular coagulation (DIC). Abdominal ultrasound and magnetic resonance imaging (MRI) revealed a large fibroid uterus with multiple masses concerning for leiomyosarcoma or leiomyoma, and transthoracic echocardiography (TTE) showed a large echo density on the tricuspid valve and a bicuspid aortic valve with mild-to-moderate stenosis. Further evaluation with transesophageal echocardiography (TEE) confirmed a large mass on the tricuspid valve and also revealed multiple echo densities on the mitral and aortic valves. Cardiac MRI (CMR) demonstrated thickened, mobile tricuspid valve leaflets with moderate regurgitation; mild-to-moderate mitral regurgitation was also seen, along with thickening of the mitral valve leaflets. However, the nature of the masses remained unclear. Despite negative blood cultures, the clinical and imaging findings raised suspicion for non-bacterial thrombotic endocarditis (NBTE), though thrombi remained a differential consideration. Given the patient’s hypercoagulable state, a multidisciplinary team initiated anticoagulation and arranged close outpatient follow-up. A cervical lymph node biopsy subsequently confirmed metastatic, poorly differentiated carcinoma with immunohistochemistry positive for CKAE1/AE3, HMWCK, and PAX8, suggesting either recurrent thymic malignancy or a primary uterine origin. A few months later, repeat TTE showed reduced severity of mitral and tricuspid regurgitation, and the valvular masses were less apparent. The patient is currently undergoing palliative chemotherapy with carboplatin, paclitaxel, and dostarlimab, continues on lifelong anticoagulation, and receives comprehensive pain management.

This case underscores the diagnostic complexity of cardiac masses, particularly in patients with malignancy. It highlights the critical role that multimodality imaging plays in resolving complex diagnostic dilemmas and emphasizes the importance of a collaborative approach in managing such patients.

## Introduction

Cardiac masses include a wide spectrum of entities, including benign tumors, malignant neoplasms, thrombi, and infectious or non-infectious vegetations. Accurate differentiation is crucial, as management strategies vary widely, ranging from anticoagulation and surgical removal to antimicrobial treatment. However, thrombi and vegetations may appear similar on echocardiography, and these overlapping imaging features frequently pose diagnostic challenges [1].

Echocardiography remains the first-line imaging modality for detecting intracardiac masses. Transthoracic echocardiography (TTE) is widely available and effective for assessing mass size, mobility, attachment, and hemodynamic impact, but it can be limited by suboptimal visualization, especially for lesions located posteriorly within the heart. Transesophageal echocardiography (TEE) offers improved spatial resolution and sensitivity for detecting smaller or multiple lesions [2]. However, echocardiography alone cannot definitively characterize the nature of the mass.

Cardiac magnetic resonance imaging (CMR) and cardiac computed tomography (CT) offer complementary information through high-resolution tissue characterization and detailed anatomical visualization, aiding differentiation between thrombi, vegetations, and tumors. Among these, CMR is considered the noninvasive gold standard for evaluating cardiac masses due to its superior soft tissue contrast and ability to assess both structure and function [1].

Vegetations, typically associated with infective endocarditis (IE), can sometimes be identified through specific features such as mobile, irregular masses, whereas thrombi tend to be more stable and have different clinical presentations. Non-bacterial thrombotic endocarditis (NBTE) is characterized by sterile vegetations composed of fibrin and platelet aggregates and is increasingly recognized in patients with advanced malignancy or hypercoagulable states [3]. In cases like ours, where echodensities are present on the tricuspid and aortic valves, it is important to consider not only the imaging findings but also the overall clinical context.

## Case presentation

**Figure 1:**
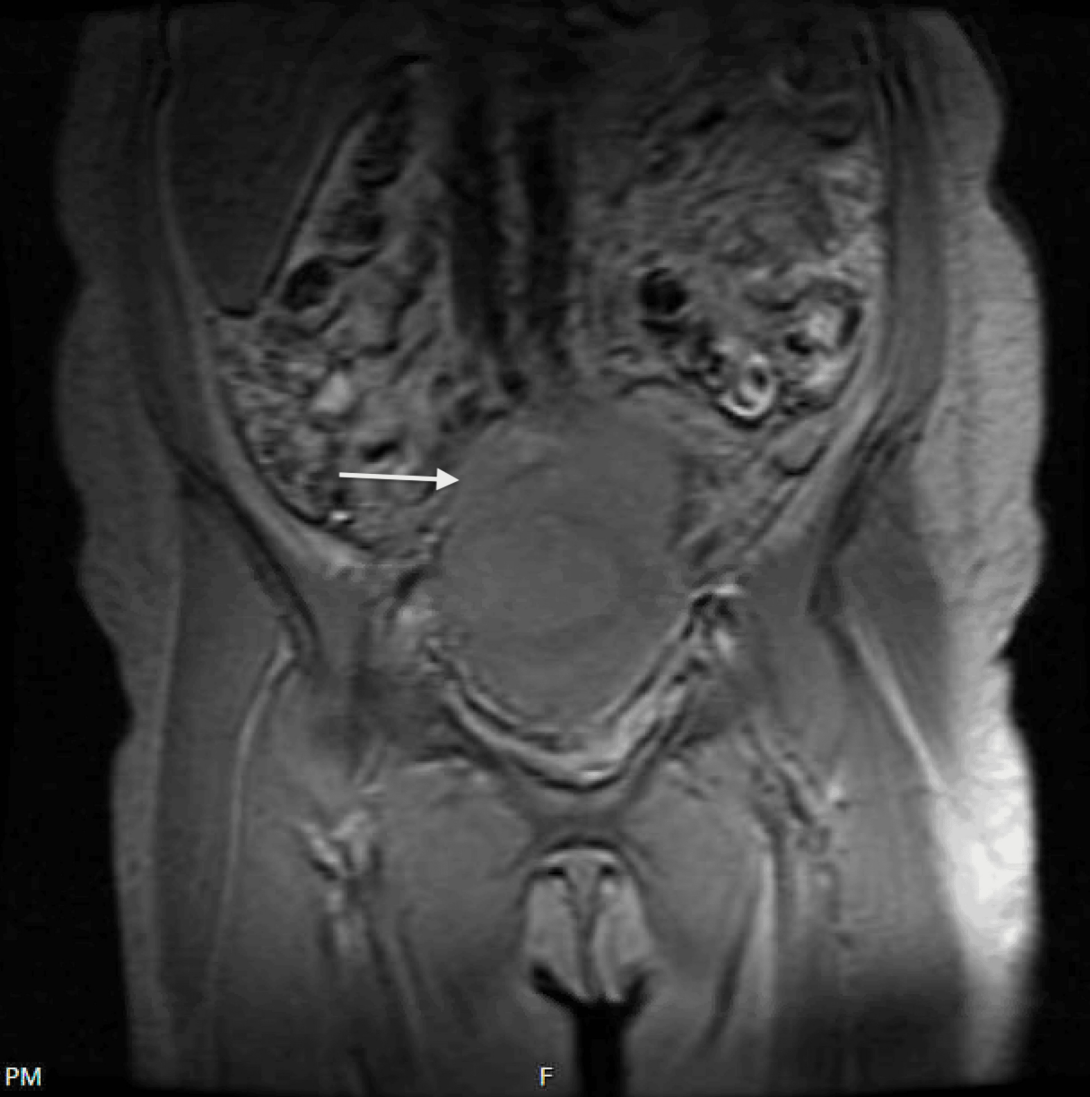
MRI of the pelvis showing a large fibroid uterus. The white arrow shows a mass in the uterus concerning for leiomyosarcoma or leiomyoma. MRI, magnetic resonance imaging

A 57-year-old postmenopausal female presented with abdominal pain, decreased appetite, and unintentional weight loss of 10-15 pounds in the last month. The patient had a history of thymic cancer status post-chemoradiation and partial thyroidectomy 11 years ago. Three months before presentation, the patient had a pulmonary embolism (PE) that was thought to be secondary to long travel. The patient was discharged after placement of an inferior vena cava (IVC) filter and initiation of rivaroxaban. A few months later, the patient started to experience abdominal pain and constipation. Abdominal ultrasound and magnetic resonance imaging (MRI) revealed a large fibroid uterus with multiple masses, concerning for leiomyosarcoma or leiomyoma (Figure 1).

The patient was supposed to follow up with a gynecologist (OB/GYN), but within a few days, she developed worsening abdominal pain and came to the emergency department for further evaluation. She was found to be hypertensive, with a blood pressure of 148/84 mmHg. On physical examination, the patient was a middle-aged woman in distress, with a normal heart rate and regular rhythm, a chest clear to auscultation, and an abdomen that was flat, soft, and tender to palpation in the suprapubic region and left lower quadrant, with positive bowel sounds. No pitting edema was appreciated in the lower extremities.

Initial blood work was concerning for disseminated intravascular coagulation (DIC) and acute end-organ dysfunction, as the patient was noted to have thrombocytopenia, decreased fibrinogen, elevated D-dimer, and fibrin split products. She also had acute anemia, deranged liver enzymes, lactic acidosis, and elevated LDH and uric acid levels. Laboratory results are summarized in Table 1.

**Table 1:** Initial laboratory findings. AST, aspartate aminotransferase; ALT, alanine aminotransferase; LDH, lactate dehydrogenase

Laboratory Assay	Value	Reference Range
Platelet count (/μL)	46,000	150,000-400,000
Fibrinogen (mg/dL)	77	200-400
D-dimer (μg/mL)	23.58	<0.50
Fibrin split products (μg/mL)	>20	<10
Hemoglobin (g/dL)	10.4	12.0-16.0
Hematocrit (%)	32.6	36-46
Total bilirubin (mg/dL)	1.3	0.1-1.2
AST (U/L)	448	10-40
ALT (U/L)	541	7-56
Lactate (mmol/L)	2	0.5-2.2
LDH (U/L)	1396	110-240
Uric acid (mg/dL)	7.4	2.4-6.0

Given the low fibrinogen, she was started on a unit of cryoprecipitate. CT of the abdomen and pelvis was performed and revealed a splenic infarct, a right lower pole renal infarct, and progression of lower extremity DVT up to the tip of the IVC filter, partially obstructing the left renal vein. After consultation with hematology, oncology, and OB/GYN, the patient was transferred to the tertiary care center for further investigations and management.

**Figure 2:**
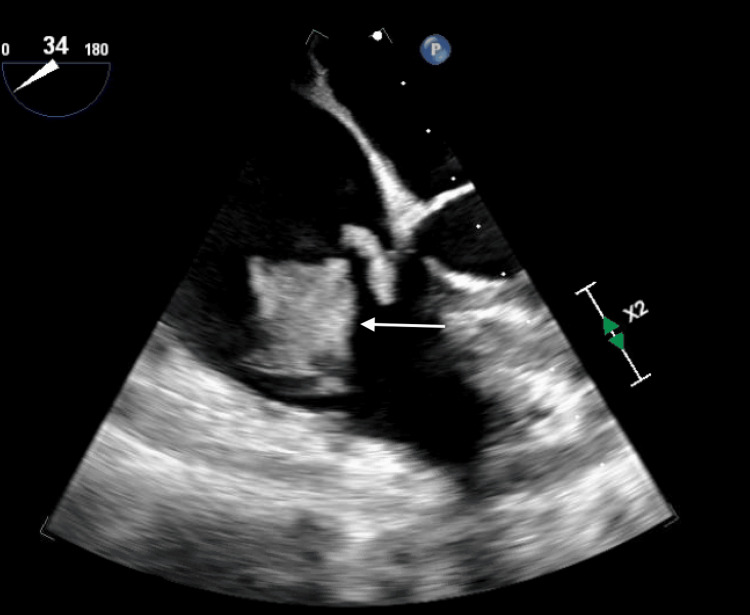
TEE showing a large mass on the tricuspid valve. The white arrow shows mass on the tricuspid valve. TEE, transesophageal echocardiogram

**Figure 3:**
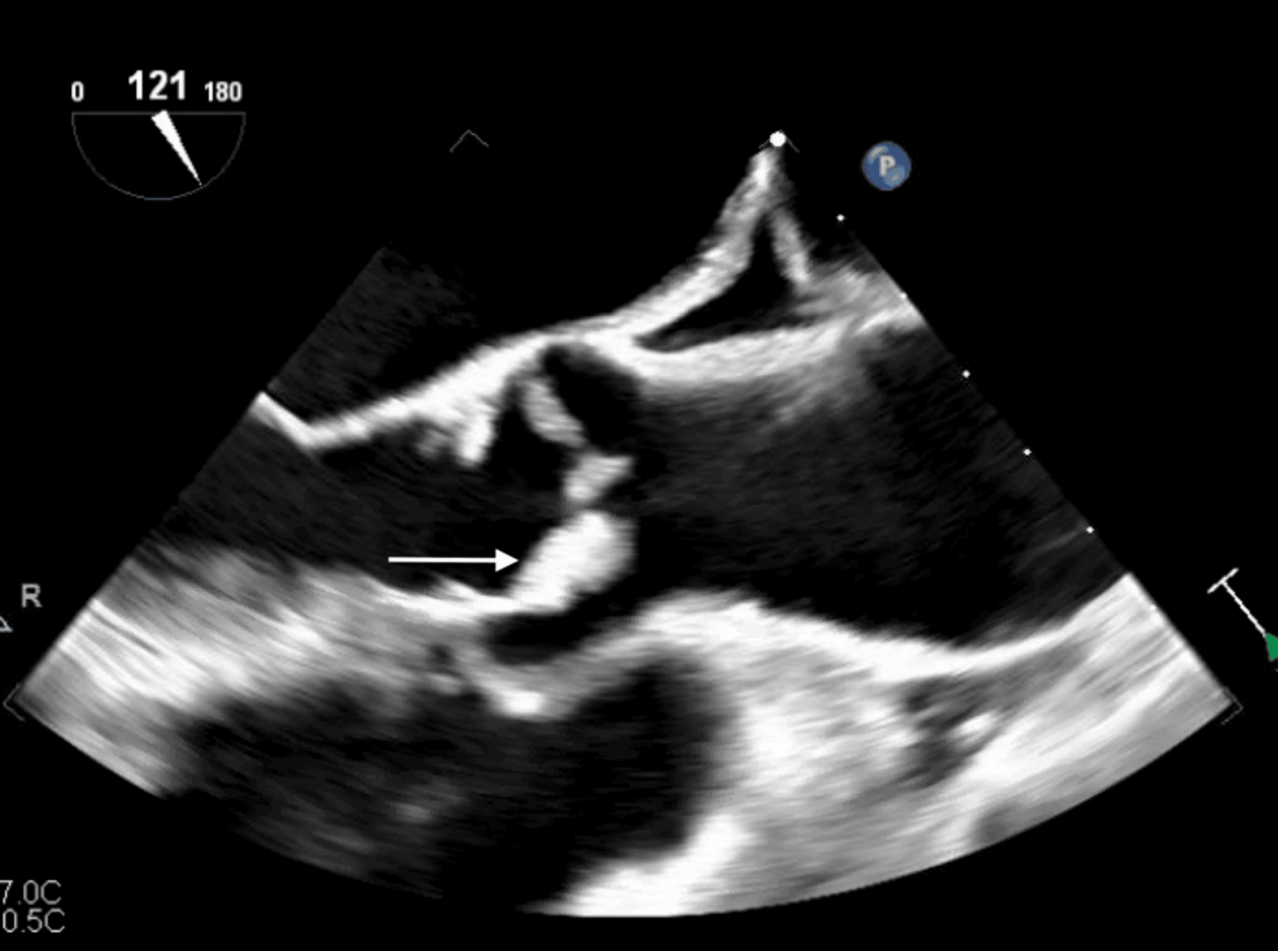
TEE image showing thickening of the aortic valve leaflets. The white arrow shows thickening of the aortic valve leaflets. TEE, transesophageal echocardiogram

**Figure 4:**
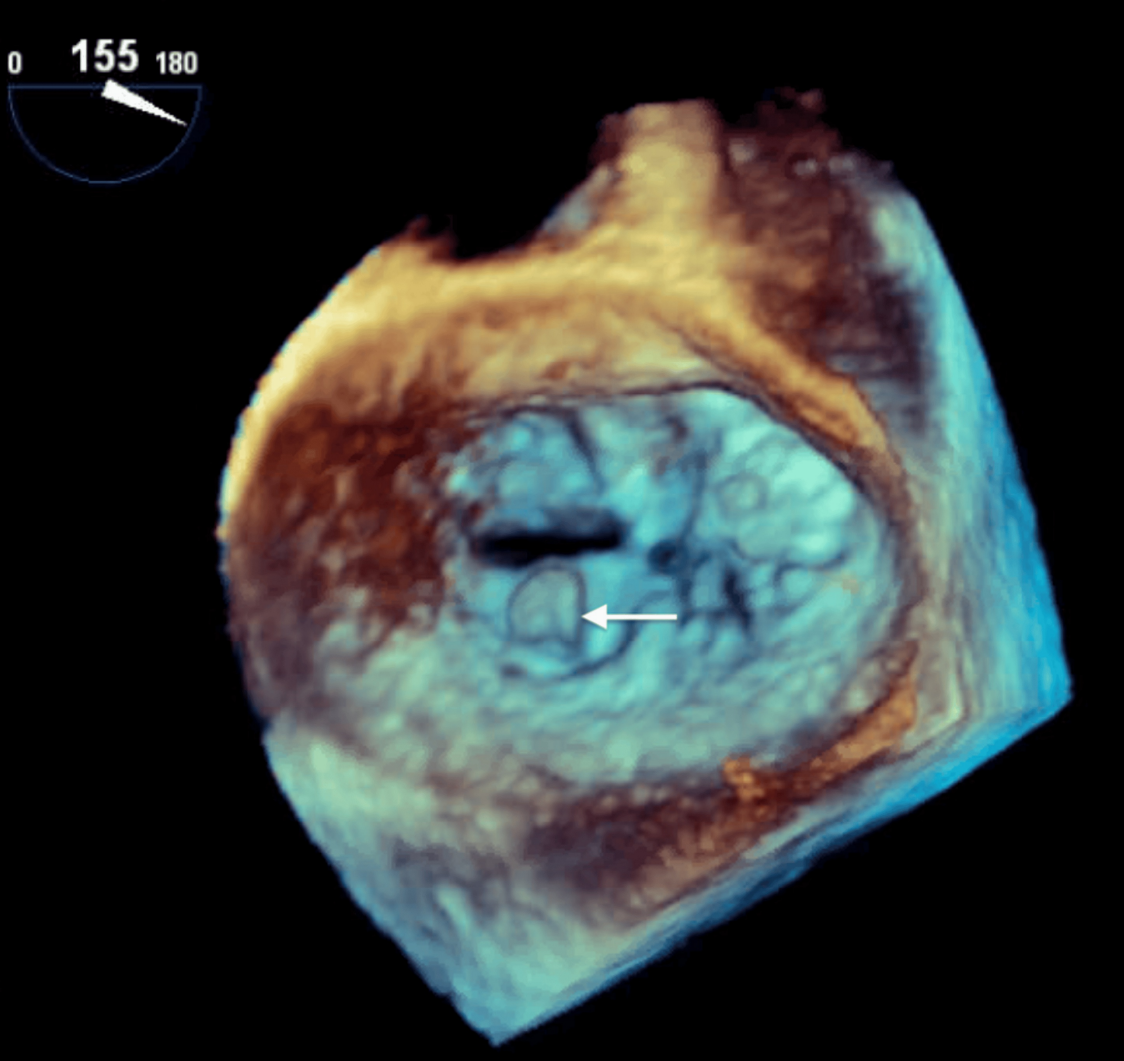
Three-dimensional echocardiogram demonstrating a mass on the mitral valve. The white arrow shows mass on the mitral valve.

Upon presentation to the tertiary care center, the patient was found to have elevated hs-troponin (275 ng/L) and proBNP (3548 pg/mL). A limited TTE was performed and showed an ejection fraction (EF) of 50%-55% and a small pericardial effusion, with no evidence of cardiac tamponade. The next day, a comprehensive TTE was performed and revealed a large echo density on the tricuspid valve with mild-to-moderate regurgitation and a bicuspid aortic valve with mild-to-moderate stenosis. In the setting of the recently identified uterine mass, differential diagnoses for the echodensities included vegetation, thrombus, or malignancy. To further evaluate these findings, a TEE was performed, which not only confirmed a large mass on the tricuspid valve but also revealed multiple echodensities on the mitral and aortic valves (Figures 2-4).

**Figure 5:**
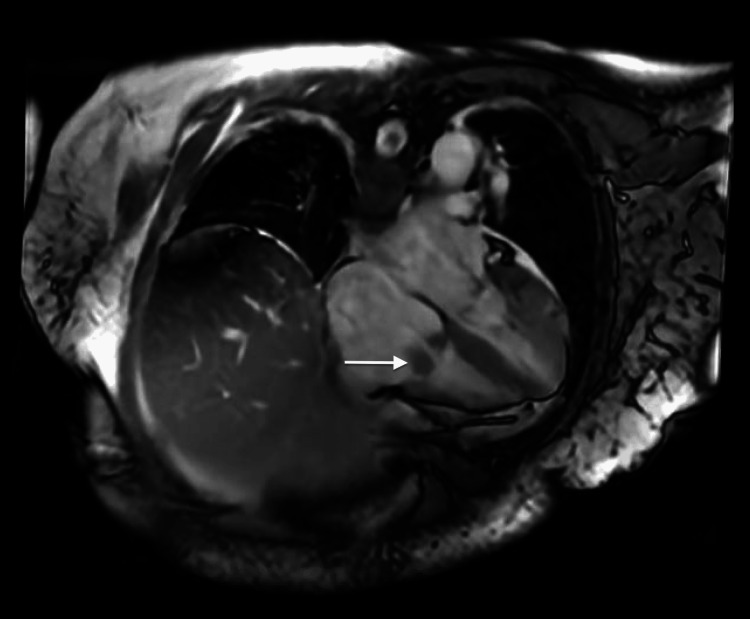
CMR showing irregular thickening of the tricuspid valve leaflets. The white arrow indicates irregular thickening of the tricuspid valve leaflets. CMR, cardiac magnetic resonance imaging

A patent foramen ovale with right-to-left shunting was also found by color Doppler. The patient was continued on a heparin drip, and blood cultures were sent, which later resulted in negative. A multidisciplinary approach was taken, including infectious disease (ID), cardiology, oncology, gynecology, and internal medicine. The patient underwent CMR, which revealed thickening and irregularity of the tricuspid valve leaflets with associated moderate tricuspid regurgitation (Figure 5).

Mild-to-moderate mitral regurgitation was also seen, with thickening of the mitral valve leaflets. A mild degree of aortic insufficiency was also noted, with abnormal thickening of the aortic valve leaflets. Unfortunately, due to the mobility of the thickened valve leaflets, the abnormal valves were difficult to further characterize on other MRI sequences. As per ID, the masses were most likely vegetations secondary to NBTE, although thrombi remained in the differential. Antibiotics were stopped, and the patient was advised to continue anticoagulation and undergo repeat TEE to re-evaluate and plan for a hysterectomy as an outpatient. The patient was discharged with follow-up arranged with oncology, cardiology, and OB/GYN.

**Figure 6:**
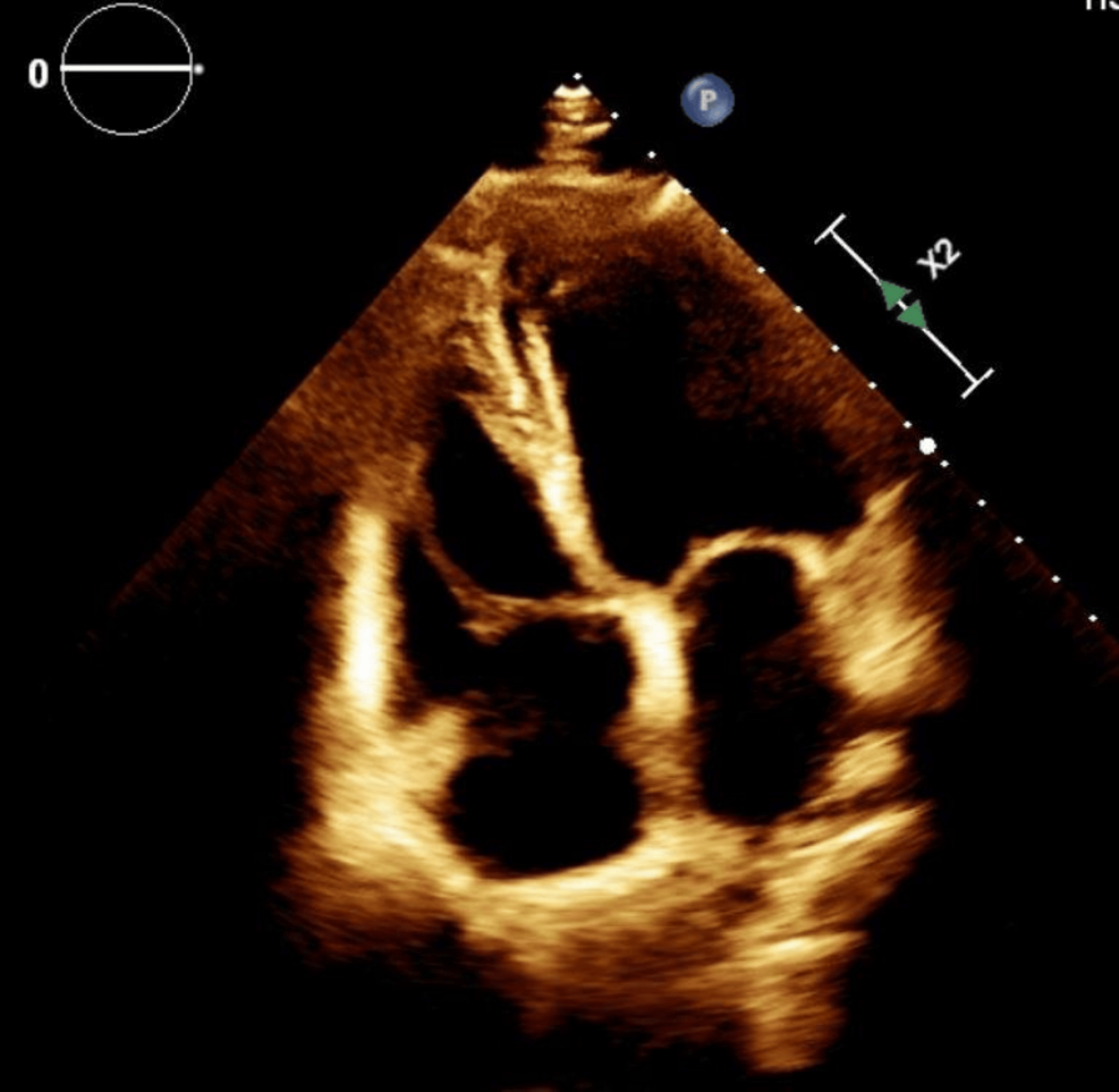
Follow-up TTE showing the resolution of mitral valve mass. TTE, transthoracic echocardiogram

As an outpatient, the patient underwent a cervical lymph node biopsy, which revealed metastatic, poorly differentiated carcinoma, and it was unclear whether this was secondary to recurrence of a thymic tumor or a primary uterine malignancy. Her tumor was positive for CKAE1/AE3, HMWCK, and PAX8, with patchy CEA/MOC-31 expression. A few months later, she underwent repeat TTE, which revealed improvement in the severity of mitral and tricuspid regurgitation, and the echodensities on these valves were less apparent (Figure 6).

The patient is maintaining close follow-up with an oncologist and is currently on palliative chemotherapy with carboplatin, paclitaxel, and dostarlimab. She will continue lifelong anticoagulation and is receiving optimal pain management.

## Discussion

Cardiac masses, whether incidental or symptomatic, are commonly found in echocardiographic imaging but can pose significant diagnostic challenges when no surgical or pathological intervention is performed. These masses remain an unsolved dilemma, as the exact nature - whether thrombus, vegetation, or other pathology - can be difficult to determine without diagnostic histologic examination [2]. Timely and accurate identification is crucial because incorrect treatment can have severe and potentially fatal consequences. For instance, anticoagulation of a vegetation misdiagnosed as thrombus may increase the risk of embolic stroke or intracerebral hemorrhage, while withholding antibiotics from a true infective vegetation can result in sepsis, valve destruction, or systemic embolization [4]. Conversely, treating a sterile thrombus with prolonged antibiotics exposes the patient to unnecessary risks, such as drug toxicity and antibiotic resistance, without therapeutic benefit. Although clinical context and laboratory findings play an important role in guiding diagnosis, challenges remain. Features such as fever, leukocytosis, positive blood cultures, and elevated inflammatory markers often point toward IE [5]. In contrast, thrombi, especially NBTE, are more commonly associated with underlying malignancy, autoimmune conditions, or hypercoagulable states and typically lack signs of infection [6]. However, this case exemplifies the complexity of diagnosing and managing cardiac masses, particularly when clinical data alone provide a broad differential diagnosis. It also highlights the importance of multimodality imaging in narrowing these differentials and guiding management decisions effectively.

A review of the literature emphasizes the critical role of multimodality imaging, particularly the integration of echocardiography, cardiac CT, CMR, and 18F-fluorodeoxyglucose positron emission tomography/CT (18F-FDG PET/CT) in accurately distinguishing between thrombus and vegetation, two common causes of cardiac masses [5]. One of the most useful imaging techniques is CMR. Through the use of late gadolinium enhancement (LGE), CMR can help identify endocardial or marginal mass enhancement, which is a hallmark of inflammation seen in endocarditis [7]. This is a key feature that can point toward vegetation, often associated with IE. In contrast, thrombi usually show little or no LGE and exhibit changes in signal intensity that further help distinguish them from vegetation.

However, while CMR is highly useful, it has limitations, particularly in terms of lower temporal resolution. In areas of accelerated blood flow, such as around heart valves, clear differentiation of masses can be challenging. In such cases, cardiac CT, especially when synchronized with the patient’s ECG, can provide superior spatial resolution and detailed imaging, making it an invaluable tool for evaluating valve-related masses [8]. Additionally, 18F-FDG PET/CT imaging can provide crucial insights, especially in cases of endocarditis. Abnormal FDG uptake around prosthetic valves is highly indicative of infection, demonstrating high sensitivity and aiding in the diagnosis of endocarditis [9].

This case illustrates the real challenge of differentiating thrombus and vegetation, two conditions that may appear similar on standard imaging but require different treatments. Multimodality imaging provides a spectrum of diagnostic information, reducing the need for invasive procedures [1]. However, some cases, particularly complicated ones like the one presented, may still require a multidisciplinary team approach. In these situations, collaboration between cardiologists, radiologists, ID specialists, and other experts is necessary to ensure an accurate diagnosis and formulate an appropriate management plan while considering all possible differential diagnoses.

Overall, this case highlights the critical role multimodality imaging plays in resolving complex diagnostic dilemmas, emphasizing the importance of a collaborative approach in managing patients with cardiac masses.

## Conclusions

In conclusion, distinguishing between thrombus and vegetation in cardiac masses is a challenging but crucial task, as misdiagnosis can lead to inappropriate treatments with potentially fatal consequences. The integration of multimodality imaging - such as echocardiography, CMR, cardiac CT, and 18F-FDG PET/CT - plays a pivotal role in narrowing down differential diagnoses and providing valuable insights without resorting to invasive procedures. CMR, for example, helps identify key features like LGE that point toward endocarditis, while thrombus typically lacks these characteristics. In cases where CMR’s temporal resolution is limited, cardiac CT or 18F-FDG PET/CT may offer superior resolution, especially in areas of accelerated blood flow, such as the heart valves. However, the complexity of these cases often necessitates a multidisciplinary approach, where collaboration between cardiologists, radiologists, and other specialists is essential for formulating an accurate diagnosis and comprehensive management plan. Ultimately, this case highlights the importance of combining clinical data, advanced imaging techniques, and expert collaboration to achieve the best outcomes for patients with cardiac masses.
